# Effect of Suspended Particulate Matter on the Accumulation of Dissolved Diarrhetic Shellfish Toxins by Mussels (*Mytilus galloprovincialis*) under Laboratory Conditions

**DOI:** 10.3390/toxins10070273

**Published:** 2018-07-03

**Authors:** Aifeng Li, Meihui Li, Jiangbing Qiu, Jialiang Song, Ying Ji, Yang Hu, Shuqin Wang, Yijia Che

**Affiliations:** 1 College of Environmental Science and Engineering, Ocean University of China, Qingdao 266100, China; limeihui02110118@163.com (M.L.); asttl@ouc.edu.cn (J.Q.); sjl0320@163.com (J.S.); jiying2018@163.com (Y.J.); ouc_huyang@163.com (Y.H.); long052193@163.com (S.W.); cheyj0808@163.com (Y.C.); 2 Key Laboratory of Marine Environment and Ecology, Ocean University of China, Ministry of Education, Qingdao 266100, China

**Keywords:** diarrhetic shellfish toxins (DST), *Mytilus galloprovincialis*, DST accumulation, DST esterification, suspended particulate matter (SPM)

## Abstract

In recent years, detection of trace amounts of dissolved lipophilic phycotoxins in coastal waters has been possible using solid phase adsorption toxin tracking (SPATT) samplers. To explore the contribution of dissolved diarrhetic shellfish toxins (DST) to the accumulation of toxins by cultivated bivalves, mussels (*Mytilus galloprovincialis*) were exposed to different concentrations of purified okadaic acid (OA) and dinophysistoxin-1 (DTX1) in filtered (0.45 µm) seawater for 96 h. Accumulation and esterification of DST by mussels under different experimental conditions, including with and without the addition of the food microalga *Isochrysis galbana*, and with the addition of different size-fractions of suspended particulate matter (SPM) (<75 µm, 75–150 µm, 150–250 µm) were compared. Results showed that mussels accumulated similar amounts of OA and DTX1 from seawater with or without food microalgae present, and slightly lower amounts when SPM particles were added. Mussels preferentially accumulated OA over DTX1 in all treatments. The efficiency of the mussel’s accumulation of OA and DTX1 from seawater spiked with low concentrations of toxins was higher than that in seawater with high toxin levels. A large proportion of OA (86–94%) and DTX1 (65–82%) was esterified to DTX3 by mussels in all treatments. The proportion of *I. galbana* cells cleared by mussels was markedly inhibited by dissolved OA and DTX1 (OA 9.2 µg L^−1^, DTX1 13.2 µg L^−1^) in seawater. Distribution of total OA and DTX1 accumulated in the mussel tissues ranked in all treatments as follows: digestive gland > gills > mantle > residual tissues. However, the percentage of total DST in the digestive gland of mussels in filtered seawater (67%) was higher than with the addition of SPM particles (75–150 µm) (51%), whereas the gills showed the opposite trend in filtered seawater with (27%) and without (14.4%) SPM particles. Results presented here will improve our understanding of the mechanisms of DST accumulation by bivalves in marine aquaculture environments.

## 1. Introduction

**Figure 1:**
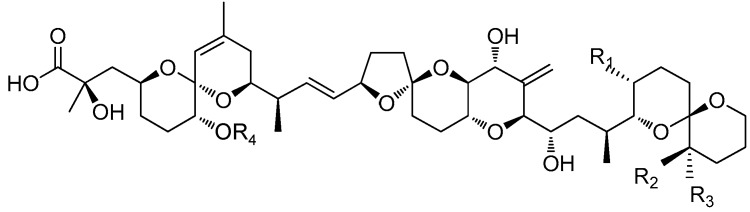
Chemical structure of okadaic acid (OA) and its derivatives. DST: diarrhetic shellfish toxins.

Okadaic acid (OA), dinophysistoxin-1 (DTX1) and -2 (DTX2) (Figure 1) are produced by some benthic dinoflagellates of the genus *Prorocentrum*, such as *P. lima* [1], *P. concavum* [2], *P. hoffmannianum* [3], *P. rhathymum* [4] and *P. foraminosum* [5], and by planktonic dinoflagellates of the genus *Dinophysis*, including *D. acuminata*, *D. acuta*, *D. caudata*, *D. fortii*, *D. miles*, *D. norvegica*, *D. ovum*, *D. sacculus* and *D. tripos* [6]. These phycotoxins are called diarrhetic shellfish toxins (DST) because when transferred to consumers through common seafood vectors, including mussels, clams, scallops and oysters [7,8], they cause severe diarrhea due to strong inhibition of serine/threonine protein phosphatase activity leading to severe mucosal damage of the intestinal tract [9]. Diarrhetic shellfish poisoning (DSP) events are a world-wide phenomenon [10,11,12,13,14,15]. The first confirmed cases of DSP in China occurred in 2011 when more than 200 residents became ill after consuming mussels (*Mytilus galloprovincialis*) harvested from coastal waters of the East China Sea [11]. DSP has been recognized as one of the five most common illnesses caused by harmful algal bloom toxins, which also include ciguatera fish poisoning, paralytic shellfish poisoning, neurotoxic shellfish poisoning and amnesic shellfish poisoning [16].

The free forms of DST (OA, DTX1 and DTX2) produced by microalgae can be esterified in many bivalve species with different fatty acids through the –OH group at the *C*-7 site [11,17,18]. These 7-*O*-acyl-OA/DTX1 esters, known as dinophysistoxin-3 (DTX3) (Figure 1), with various molecular weight and fatty acid chain-lengths ranging from 12 to 22 carbons, were stored mainly in the digestive gland of scallops in a previous study [19]. Diverse DTX3 components also play important roles in the intoxication of human consumers [20,21] because they can be hydrolyzed by lipases and other enzymes to release toxins into the gastrointestinal tract [22,23,24]. Some other diol-esters are biosynthesized by esterification of the *C*-1 acid group with 4-10-carbon side chains in the dinoflagellates *P. lima* and *D. acuta* [25]. Alkaline hydrolysis is usually used to release free toxin forms from DTX3 in order to accurately quantify the potential DST levels in seafood products. Currently, a regulatory limit of 160 µg OA eq. kg^−1^ for OA and its analogues in shellfish meat is implemented by the European Union, but a more rigid control, 45 µg OA eq. kg^−1^, is recommended by the European Food Safety Authority [26].

A great deal of effort has been devoted to forecasting toxic blooms caused by DST-producing microalgae and protecting human health. Many countries with a well-developed shellfish industry have implemented regular monitoring programs, including monitoring density of microalgae in seawater and toxin contamination of shellfish tissues, as well as detection of toxins in seawater using solid phase adsorption toxin tracking (SPATT) or solid phase extraction (SPE) methods. The SPATT technology was first adopted by MacKenzie et al. [27] to monitor dissolved lipophilic toxins in seawater and is considered an effective complementary tool for monitoring and studying algal toxin dynamics in the field and in laboratory experiments [28,29,30]. In addition, SPATT resins have shown many advantages in the sample preparation process [31]. Nevertheless, some SPATT-based monitoring results have not supported its value as an early warning tool for shellfish contamination with DST [32,33]. In recent years, solid phase extraction (SPE) cartridges have been used to sample dissolved lipophilic toxins in seawater. The adsorbed toxins were analyzed with highly sensitive detection technologies such as liquid chromatography-tandem mass spectrometry (LC-MS/MS), and trace amounts of DST were detected in most samples from coastal waters of Qingdao, China, since October 2012 [34,35]. This dissolved DST fraction may contribute to the accumulation of these toxins by the bivalves exposed to them. 

In the present study, accumulation of dissolved DST by mussels was simulated in the laboratory and the effect of suspended particulate matter (SPM) on toxin accumulation was explored. In addition, biotransformation processes and the distribution of DST in various mussel tissues were also examined.

## 2. Results

### 2.1. Accumulation of Dissolved OA and DTX1 from Seawater by Mussels

**Figure 2:**
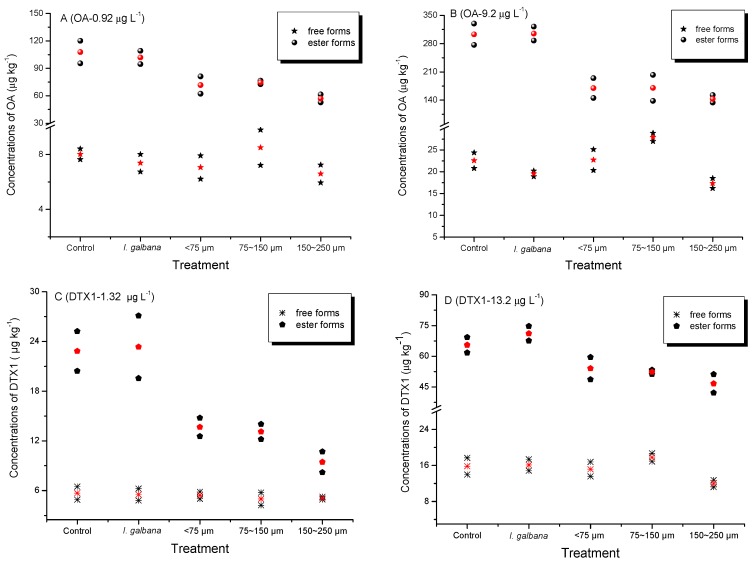
Concentrations of OA (**A**,**B**) and DTX1 (**C**,**D**) accumulated by mussels from seawater for treatments spiked with low (**A**,**C**) and high (**B**,**D**) toxin concentrations. Control: No microalgae or suspended particulate matter (SPM) added; either *I. galbana* or one of three different particle size-fractions of SPM (<75 µm, 75–150 µm, and 150–250 µm) were added for the treatments; red symbols indicate mean values of duplicate treatments.

Accumulation of dissolved OA and DTX1 by mussels was observed in all treatments. Concentrations of free and ester forms of the toxins are shown in Figure 2. Trace amounts of OA (~7–8 µg kg^−1^) and DTX1 (~6 µg kg^−1^) were detected in the whole soft tissue of mussels exposed to low toxin levels, and about three times more in mussels exposed to the high levels. Different accumulation efficiencies of OA and DTX1 occurred in mussels exposed to various toxin concentrations (Table 1). Similar proportions of esterified toxins were estimated for OA or DTX1 accumulated by mussels exposed to different toxin concentrations, although some slight discrepancies were noted (Table 2).

### 2.2. Effect of OA and DTX1 on the Feeding Ability of Mussels

**Figure 3:**
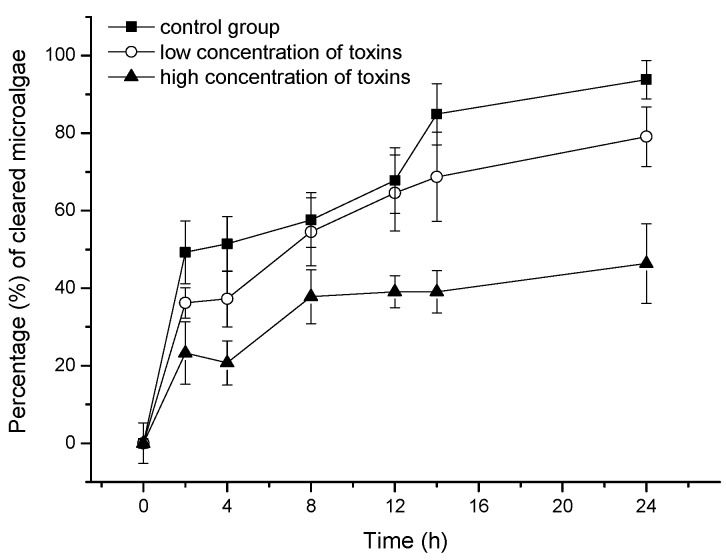
Percentage of microalgae cleared by mussels subject to different concentrations of dissolved toxins. Control: no toxins added; low toxin concentrations: 0.92 µg L^−1^ OA and 1.32 µg L^−1^ DTX1; high toxin concentrations: 9.2 µg L^−1^ OA and 13.2 µg L^−1^ DTX1.

Although mussels were able to accumulate dissolved OA and DTX1, the toxins negatively affected their ability to feed on the microalga, *Isochrysis galbana*. The proportions of microalgae cleared by mussels subject to different treatments are shown in Figure 3.

### 2.3. Tissue Distribution of Toxins Accumulated by Mussels

**Figure 4:**
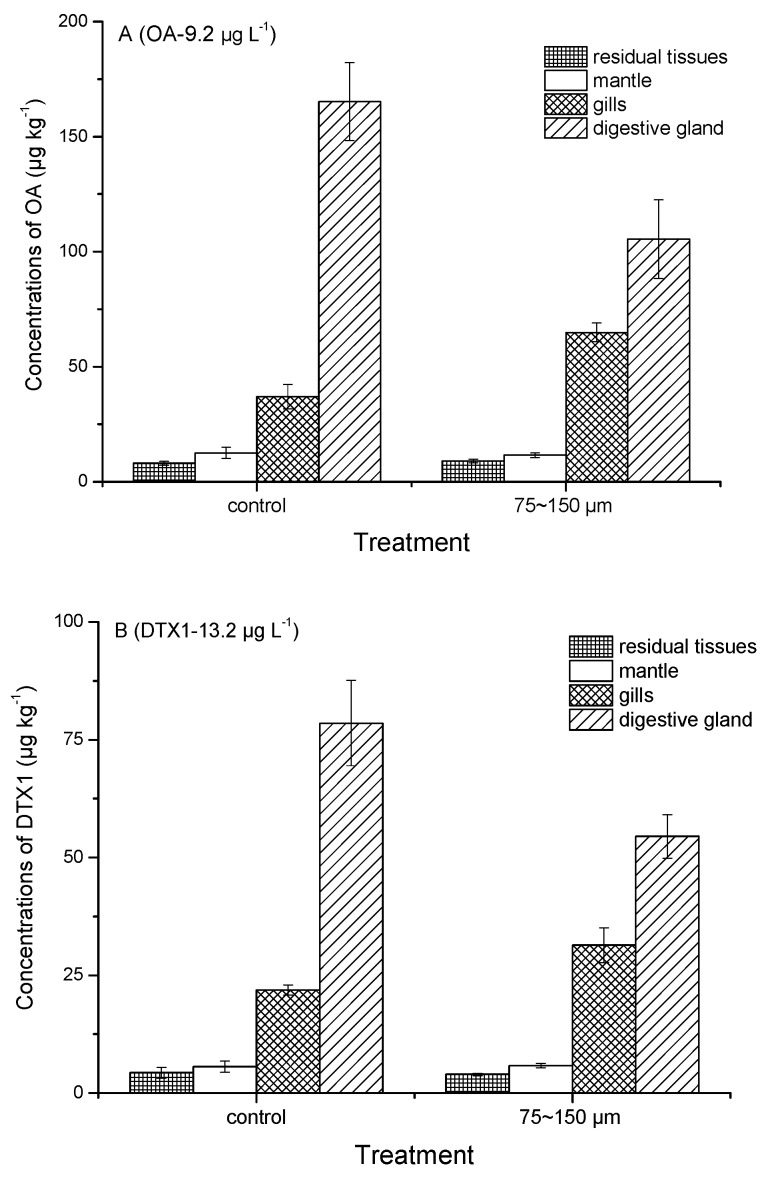
Distribution of OA and DTX1 toxins in different tissues of mussels exposed to high concentration of dissolved toxins (OA-9.2 µg L^−1^ (**A**) and DTX1-13.2 µg L^−1^ (**B**)) in the presence and absence of SPM particles (75–150 µm).

**Figure 5:**
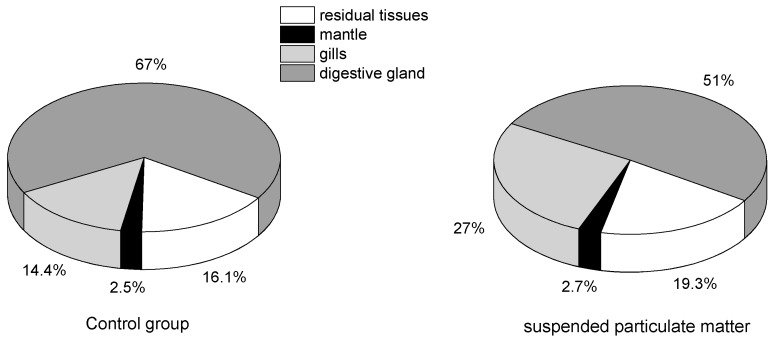
Percentage of toxins accumulated in different mussel tissues in the absence or presence of suspended particulate matter particles.

Tissue distribution of OA and DTX1 accumulated by mussels with or without the addition of SPM particles were compared. Concentrations of OA and DTX1 in mantle, gills, digestive gland and residual tissues are shown in Figure 4. Relative percentages of total toxin amount distributed in the different tissues are indicated in Figure 5.

## 3. Discussion

Mean residual levels of OA ranged from 2.71 to 14.06 ng L^−1^ in seawater samples collected from Jiaozhou Bay, China, in July, August, and September 2014, but no DTX1 was detected [35]. Residual OA concentrations ranged between 1.41 and 89.52 ng L^−1^ in Qingdao coastal waters from October 2012 to September 2013 [34]. Trace amounts of OA were detected in coastal areas every month, except for elevated levels observed in August (the highest concentration = 89.52 ng L^−1^) [34], indicating that OA degradation in seawater can occur slowly. Importantly, no blooms of *Dinophysis* or *Prorocentrum* were reported in Qingdao coastal waters during the entire year when seawater samples were collected and analyzed [34]. We hypothesize that the dissolved OA concentrations during blooms of DST-producing microalgae are higher than the residual levels reported previously [34,35]. In order to assess the contribution of dissolved DST to their accumulation by cultured bivalves, OA and DTX1 (molar ratio OA/DTX1 ≈ 0.71) from *P. lima* cultures were spiked into filtered (0.45 µm membrane) seawater for exposure experiments in this study. No DTX2 or DTX3 were detected in the strain of *P. lima* used here. The DST concentration used in our experiments was higher than the residual levels found in natural seawater to ensure detection of toxins accumulated by mussels after 4 days of exposure. Low levels of OA and DTX1 were set at 0.92 and 1.32 µg L^−1^, respectively, and the high levels 10-fold more. The detection of OA and DTX1 confirmed that mussels (*M. galloprovincialis*) can accumulate dissolved OA and DTX1 from seawater under all treatment conditions. A previous study showed that blue mussels (*M. edulis*) accumulated dissolved azaspiracids (AZA) to reach concentrations above the regulatory limit [36].

To our knowledge, this is the first report confirming that bivalves are able to accumulate dissolved DST from seawater. The addition of microalgae (*I. galbana*) did not improve the accumulation of dissolved DST by mussels, and SPM particles, especially the 150–250 µm size fraction, somewhat inhibited the OA and DTX1 accumulation efficiency (Table 1). According to a previous study, the clearance and ingestion rates of scallops (*Chlamys farreri*) and clams (*Ruditapes philippinarum*) increased when the SPM particle concentration increased from 20 mg L^−1^ to 50 mg L^−1^, and in mussels (*M. galloprovincialis*) when the concentration increased from 20 mg L^−1^ to 100 mg L^−1^ [37]. The concentration of SPM particles used here (30 mg L^−1^) did not inhibit the clearance or ingestion rates by mussels. The lack of obvious positive effects associated with feeding on *I. galbana* demonstrated that the energy provided by non-toxic prey did not lead to improved DST accumulation nor did the microalgal cells enhance toxin accumulation via adsorptive mechanisms. In a previous study, the total amount of AZA accumulated by mussels was also virtually identical in dissolved AZA treatments with or without the addition of the non-toxic *I.* affinis *galbana* [36]. The accumulation efficiency ratios of OA to DTX1 by mussels ranged from 5.43 to 6.55 and from 3.67 to 5.56 in seawater spiked with low and high toxin levels, respectively (Table 1), which demonstrated that although only one methyl group distinguishes OA from DTX1, dissolved OA was accumulated preferentially by mussels (Figure 1). This discrepancy was also noted in our previous field experiments carried out in the coastal waters of Qingdao, China [33], in which the amount of OA adsorbed by SPATT bags was much higher than that of DTX1, although similar concentrations of OA and DTX1 were obtained by SPE cartridges. The OA content measured in scallops was also higher than for DTX1 [33]. To our knowledge, the difference in OA versus DTX1 accumulation by bivalves was not identified in previous studies due to a focus on DST accumulation by shellfish through feeding on toxic microalgae [19,38]. In the present study, the accumulation efficiencies of OA and DTX1 in mussels decreased sharply in the high toxin level treatment (Table 1). A possible explanation is that OA and DTX1 inhibited the filtration ability of mussels. That was the case in feeding experiments with an AZA-producing microalga (*Azadinium spinosum*), which had a negative effect on mussel filtration compared to non-toxic microalgal prey (*I.* aff. *galbana*) [39]. The proportion of *I. galbana* cells cleared by mussels in the current study decreased significantly over the 24 h feeding period in seawater containing dissolved DST (Figure 3). Moreover, it was confirmed that dissolved OA and DTX1 inhibited the mussel filtration ability. It can be expected that cultured bivalves exposed to blooms of DST-producing microalgae will be affected in a similar way. Although bivalves can survive DST contamination, their physiological condition and nutritional status will likely be adversely affected.

Fatty acid esters of OA and DTX1, collectively known as DTX3 and frequently found in bivalve field samples [11,13,17,18,21,40] were the predominant toxins accumulated by mussels in all treatments (Figure 2). Blue mussels (*M. edulis*) feeding on the toxic dinoflagellate *Dinophysis acuta* [38] and scallops (*P. yessoensis*) feeding on *D. fortii* [19] were also found to metabolize large proportions of OA, DTX1 and DTX1b to DTX3 under laboratory conditions. Esterification of OA and DTX1 was also observed in the present study in mussels after direct accumulation of these toxins dissolved in seawater. The DTX3 levels accumulated in mussels in the presence of SPM particles were lower than those observed for any of the other treatments (Figure 2). This discrepancy may reflect a possible negative effect of ingested SPM particles on the esterification process. SPM particles are usually retained by the gills and eliminated by the labial palps, which could affect the respiratory efficiency of filter-feeding mussels. Higher proportions of esterified OA as compared to those of DTX1 were also found in mussels (Table 2), which may explain the preferential accumulation of OA versus DTX1 during the exposure period. However, a similar pattern of DST tissue distribution occurred in mussels with or without SPM particles (75–150 µm) added (Figure 4). Total OA and DTX1 content in these mussels ranked as follows: digestive gland > gills > mantle > residual tissues. The proportion (%) of DST in the digestive gland was higher in the control group (67%) than in the seawater plus SPM treatment (51%), but the gills exhibited the opposite trend (Figure 5). This difference suggests that SPM particles do not facilitate enhanced accumulation of dissolved OA and DTX1 due to possible adsorption and transporter actions, but instead contribute to toxin retention in the gills. In a previous study of AZA uptake by mussels, the percentage of AZA accumulated in the digestive gland was highest in mussels fed with live *A. spinosum* cells, followed (in decreasing order) by those provided with lysed cells, dissolved AZA plus non-toxic cells, and dissolved toxins; however, a large proportion of toxins (42% or 46%) were stored in the gills when mussels accumulated dissolved AZA from seawater [36]. A dissolved AZA accumulation route through the gills during respiratory and filtration activities was hypothesized in the same study [36]. In the present study, it was expected that toxins adsorbed by SPM particles retained in the gills during the filtration process would contribute to the total amount of toxins retained in this tissue compartment. Yet, no enhancement in dissolved DST accumulation by mussels in the SPM treatments (regardless of size fraction) was observed.

DST-producing dinoflagellates, such as *Prorocentrum* spp. and *Dinophysis* spp., release cellular DST into the culture medium as part of their metabolism [41,42], and large amounts of DST have also been detected in the water column during *Dinophysis* blooms [27]. A range of trace amounts of OA have been measured in coastal waters of the Yellow Sea of China, although no blooms of *Dinophysis* or *Prorocentrum* were observed in the area during the study period [33,34,35]. Based on the new findings reported here, the persistence of trace OA concentrations in seawater will contribute to DST accumulation by bivalves. OA and DTX1 toxins accumulated by mussels and scallops feeding on toxic microalgae, except for the DTX3 stored in intracellular bodies, may be excreted into the surrounding seawater with minimal metabolic transformation [19,38]. The free forms of dissolved OA and DTX1 possibly circulated through mussels and seawater in this study. We hypothesize that DTX1 was degraded to other derivatives in the 96-h exposure period. Field investigations on lipophilic shellfish toxins in our previous study also hinted that OA in seawater was more stable than DTX1 [33]. This is consistent with the fact that OA was detected in marine sediments ranging from 0.78 to 3.34 ng g^−1^ dry weight [35]. In addition to the heterogeneous vertical distribution of *Dinophysis* cells in the water column, the accumulation of dissolved DST by mussels documented herein represents another important argument against the early warning of lipophilic shellfish toxin events based solely on dinoflagellate cell numbers [43]. Moreover, the risk of DSP outbreaks would increase if blooms of toxic *Prorocentrum* spp. occurred in marine benthic environments. We suggest that dissolved toxins should be monitored routinely and their dynamics investigated further to improve forecasting bivalve contamination with DST.

## 4. Conclusions

Accumulation of dissolved OA and DTX1 by mussels (*M. galloprovincialis*) was confirmed in laboratory experiments in filtered seawater with and without microalgal prey (*I. galbana*) and in the presence of different size-fractions of SPM. No positive effect of the food microalgae on DST accumulation efficiency was observed, but a slight negative effect of the SPM particles was noted. Higher accumulation efficiencies of OA as compared to DTX1 were recorded in all treatments, and the same was observed for both toxins in seawater spiked with low concentrations of the two toxins. Most of the accumulated OA and DTX1 was esterified to DTX3 in mussels in all treatments. The proportion of microalgal cells (*I. galbana*) cleared by mussels was inhibited by dissolved OA and DTX1 or by other compounds still present in the purified extract of *P. lima*. Total amount of OA and DTX1 accumulated in mussel tissues ranked as follows: digestive gland > gills > mantle > residual tissues, in all treatments. However, the proportion of total DST in the digestive gland in filtered seawater exceeded that in the presence of SPM particles (75–150 µm), but the opposite trend occurred in gills for the same conditions. The findings reported here will help us to improve the understanding of DST accumulation mechanisms by bivalves in the field.

## 5. Materials and Methods

### 5.1. Chemicals

Acetonitrile, methanol, monopotassium phosphate (KH_2_PO_4_) and disodium hydrogen phosphate (Na_2_HPO_4_) were obtained from Merck Ltd. (White-house Station, NJ, USA); formic acid, ammonium formate, sodium hydroxide, ammonium hydroxide, and hydrochloric acid (HCl) from Fisher Scientific (Fair Lawn, NJ, USA) and OA and DTX1 reference materials from the National Research Council of Canada (Halifax, NS, Canada), and Wako Pure Chemical Industries, Ltd. (Osaka, Japan), respectively. Milli-Q water (18.2 MΩ cm or better) was supplied by a Milli-Q water purification system (Millipore Ltd., Bedford, MA, USA).

### 5.2. Microalgae

*Prorocentrum lima* strain IP797 (Bigelow laboratory, National Center for Marine Algae and Microbiota, USA) was grown with filtered (0.45-µm mixed fiber membrane) and autoclaved (121 °C for 20 min) seawater (pH 8.0 ± 0.1, salinity 30 ± 1) enriched with *f*/2-Si medium [44] in conical 5000 mL flasks. Larger volumes of *P. lima* were grown in a photo-bioreactor (120 L) at 16 °C under the same light intensity of 111 µmol m^−2^ s^−1^ with a 12-h light: 12-h dark cycle. All the cultures were gently shaken or stirred twice per day at morning and night, respectively.

An axenic culture of *Isochrysis galbana* strain 3011 (Ocean University of China collection) was used as a non-toxic prey for mussels. Culture conditions were the same as those described above for *P. lima* cultures except for the temperature, which was set at 20 °C.

### 5.3. Toxin Extraction and Purification

Cells of *P. lima* were collected with a silk mesh (25 µm), transferred into 50 mL centrifuge tubes, centrifuged at 8000× *g* for 5 min, the supernatant was discarded and the pellet was stored at −20 °C. One g (wet weight) of cells was weighed and transferred into a 10 mL centrifuge tube, 3 mL of methanol was added and the tube was sealed before being submerged in liquid nitrogen for 15 min. Then, the cells were sonicated for 30 min at 10 °C, followed by a three-fold freeze-thaw cycle, centrifugation at 8000× *g* for 10 min and then the supernatant was transferred to a glass vial. Three mL of methanol were added into the tube and the sample was centrifuged again. This extraction process was repeated twice before the supernatants were mixed in a glass vial. The extract was filtered through a 0.22 µm membrane filter (Jinteng, Tianjin, China) and stored at −20 °C.

Salts and pigments from the toxin extracts were removed by SPE purification procedure [33]. Fifteen mL of methanol were used to activate the HLB cartridge (Oasis, 3 mL, 200 mg) and then 15 mL of 20% methanol solution was added to equilibrate the cartridge. Further 15 mL of 20% methanol solution were used to wash the cartridge after the toxin extract (3–5 mL) was loaded and 15 mL more was used to elute toxins from the SPE cartridge. The rates of the activation and equilibration steps were about 1 mL min^−1^, and of the sample loading, washing and elution about 0.5 mL min^−1^. The eluate was concentrated under N_2_ at 30 °C and filtered through a 0.22 µm organic membrane filter (Jinteng, Tianjin, China). It was stored at −20 °C until analysis. OA and DTX1 predominated the toxin profile of the purified extract [45,46] and their concentrations were quantified using a LC-MS/MS method [47]. The toxin extract was dried under N_2_ at 30 °C and the residual was re-dissolved in filtered seawater (0.45 µm) before adding to the feeding experiment.

### 5.4. Preparation of Suspended Particulate Matter

Surface sediments (<2 cm) were collected from Jiaozhou Bay (120.2573° E; 36.1796° N) and dried at room temperature (total organic carbon ~1.88%) before grinding in a mortar and sieving them through 200, 100 and 60 mesh sieves. Finally, three different size-fractions of SPM were obtained: <75 µm, 75–150 µm, and 150–250 µm, respectively [37].

### 5.5. Design of Mussel Feeding Experiments

#### 5.5.1. Effect of Suspended Particulate Matter on the Accumulation of Toxins by Mussels

Healthy looking adult mussels (*Mytilus galloprovincialis*) were obtained from Qingdao seafood market. Natural seawater was used to carefully wash and clean the shells’ surface, and the connective byssus between individuals was gently cut with scissors. Then, individual mussels were acclimated for 1 week before the experiment in filtered seawater (0.45 µm membrane, salinity 29–30, pH 8.1–8.3, temperature 15–18 °C) with continuous aeration (DO > 5 mg L^−1^). The seawater was renewed twice per day, at morning and night respectively, with no addition of microalgal prey. Five individuals were harvested randomly to analyze their background levels of OA and DTX1 before the experiment.

**Figure 6:**
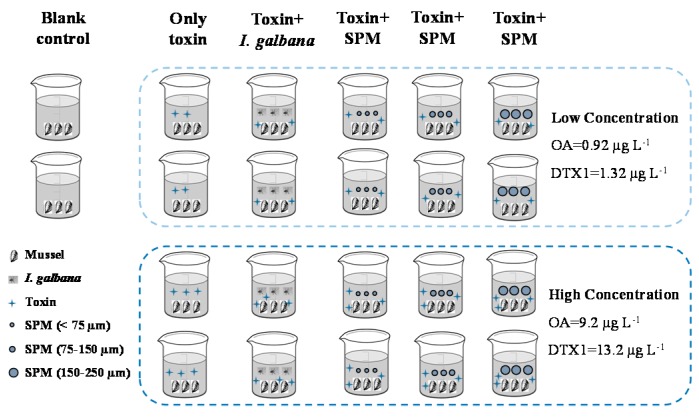
Design diagram of the exposure experiments (SPM = suspended particulate matter).

The design diagram of exposure experiments is shown in Figure 6. A total of 22 glass beakers (5 L) were filled with filtered seawater (0.45 µm) and three mussels were added per beaker. Cells of *I. galbana* were collected at the exponential growth phase, and three different SPM (<75 µm, 75–150 µm, 150–250 µm) size-fractions were added to each of the four beakers, with an initial microalgal density of about 1 × 10^6^ cells L^−1^ and a SPM concentration of 30 mg L^−1^. Then the mixture of OA and DTX1 was added to these 16 beakers in two different concentrations of toxins (low: OA 0.92 µg L^−1^, DTX1 1.32 µg L^−1^; high: OA 9.2 µg L^−1^, DTX1 13.2 µg L^−1^) to compare the effects in duplicate treatments. Both concentration levels of toxins were also added to four controls without microalgae and SPM, in duplicate treatments. Only filtered seawater and mussels were in the other two beakers as blank control treatments. All mussels were cultured for 4 days under the same conditions before terminating the feeding experiment. The ratio of the total OA or DTX1 accumulated by mussels to the total amount of toxins added into the beaker was calculated as the accumulation efficiency of the toxins shown in Table 1. The accumulation efficiency was calculated as the ratio between the amount of toxins in the mussels and that supplied in the water.

#### 5.5.2. Effect of Toxins on Mussels Feeding Behaviors

A total of nine glass beakers (5 L) were filled with filtered seawater (0.45 µm) and cells of *I. galbana*, collected at the exponential growth phase. The initial density of microalgae was 1.83 × 10^6^ cells L^−1^ to start the experiment. Three healthy mussels were placed in each beaker. Then two different concentrations of toxins (low: OA 0.92 µg L^−1^, DTX1 1.32 µg L^−1^; high: OA 9.2 µg L^−1^, DTX1 13.2 µg L^−1^) were added to the beakers in triplicate, respectively, and no toxins were added in the other three beakers. Microalgal density in all beakers was counted throughout the entire feeding period. The proportion of *I. galbana* cells cleared by mussels was calculated as the ratio between the number of algal cells eaten and the initial amount of microalgae added to the beakers.

#### 5.5.3. Esterification and Distribution of OA and DTX1 in Mussels

Three healthy mussels were placed in each glass beaker (5-L) filled with filtered seawater (0.45 µm). The same density of microalgae (*I. galbana*; ~1 × 10^6^ cells L^−1^) was added to six glass beakers. OA and DTX1 toxins (OA 9.2 µg L^−1^, DTX1 13.2 µg L^−1^) were added into three of these beakers and no toxins were added to the other three. SPM (size-fraction 75–150 µm, 30 mg L^−1^) was added to three glass beakers and the same concentration of toxins was also spiked. The mussels were taken out and different parts including gills, mantle, digestive gland and residual tissues were dissected after four days of feeding. Free and esterified forms of OA and DTX1 in the different mussel tissues were analyzed.

### 5.6. Extraction of Toxins in Mussels

Free toxin forms were extracted from mussel tissues according to Li et al. [11]. In brief, 1 g of homogenized tissue and 3 mL of methanol added to a 10-mL centrifuge tube were mixed with a vortex, the mixture centrifuged at 8000× *g* for 10 min and the supernatant was transferred to a 10-mL volumetric flask. Three mL of methanol was added and extracted twice, and all supernatants combined in the volumetric tube. Finally, the extract was made up to scale using methanol and the extraction ratio was 1 g: 10 mL. One mL of extract was filtered (0.22 µm membrane filter) and stored in sample vials at −20 °C until analysis.

The esterified forms of OA and DTX1 toxins were analyzed following [48]. One mL of the filtered (0.22 µm) extract of free toxin forms was transferred to a 4-mL glass vial, and 125 µL of 2.5 M NaOH solution was added and mixed. Then the sealed mixture was hydrolyzed at 76 °C for 40 min, neutralized with 125 µL of 2.5 M HCl and kept at room temperature. One mL of chloroform was used for a liquid-liquid extraction for the hydrolyzed extract, and this process was repeated. Finally, the chloroform phase was dried under N_2_ at 40 °C. The residual material was suspended in 1 mL of methanol, which was filtered (0.22 µm membrane filter) and stored in a sample vial at −20 °C.

### 5.7. LC-MS/MS Analysis of OA and DTX1 Toxins

An Agilent 6430 tandem quadrupole mass spectrometer coupled with an Agilent 1290 HPLC (Palo Alto, CA, USA) was used with an ESI interface. An X-Bridge™ C18 column (150 × 3 mm i.d, 5 mm, Waters, Milford, MA, USA) at 35 °C was used to separate OA and DTX1 toxins. The alkaline elution phase (pH = 11) was composed by mobile phases A (water) and B (90% acetonitrile) both containing 6.7 mM NH_4_OH [49]. A gradient was run at 300 µL min^−1^ starting with 10% ‘B’ for 1 min, and increasing linearly to 90% ‘B’ over 9 min. The mobile phase was held at 90% ‘B’ for 3 min, returned to 10% ‘B’ over 2 min, and held for 3 min before re-equilibration for the next run. An injection volume of 5 µL was adopted here.

The atomization device press was set at 40 psi, and the capillary voltage was 4000 V. The temperature of ESI source and dry N_2_ gas (flow rate 10 L min^−1^) was set at 110 °C and 350 °C, respectively. OA and DTX1 toxins were qualified and quantified by the selective reaction monitoring mode of the negative mode, and the transition ions *m*/*z* 803.5 -> 255.2, 151.1 (OA), and *m*/*z* 817.5 -> 255.2, 151.1 (DTX1), respectively. OA and DTX1 were quantified by comparing their peak areas with those of solutions with a known concentration.

## Figures and Tables

**Table:** 

DST	R_1_	R_2_	R_3_	R_4_	Molecular Weight
**OA**	CH_3_	H	H	H	804.5
**DTX1**	CH_3_	CH_3_	H	H	818.5
**DTX2**	H	H	CH_3_	H	804.5
**DTX3**	H or CH_3_	H or CH_3_	H or CH_3_	Acyl	1014~1082

**Table 1:** Percentage (%) of esterified OA and DTX1 in mussels subject to different treatments.

Treatments	OA (µg L^−1^)		DTX1 (µg L^−1^)		OA:DTX1	
	0.92	9.2	1.32	13.2	Low Toxin Level	High Toxin Level
Control	53	15	9.1	2.7	5.82	5.56
*Isochrysis galbana*	50	15	9.2	2.9	5.43	5.17
SPM < 75 μm	36	8.8	6.1	2.4	5.90	3.67
SPM 75–150 μm	38	9.1	5.8	2.4	6.55	3.79
SPM 150–250 μm	29	7.4	4.6	2.0	6.30	3.70

Control: filtered seawater with no microalgae or suspended particulate matter (SPM) added.

**Table 2:** Proportions (%) of OA and DTX1 esterified by mussels under different treatments.

Treatments	OA (µg L^−1^)		DTX1 (µg L^−1^)	
	0.92	9.2	1.32	13.2
Control	93	93	80	81
*Isochrysis galbana*	93	94	81	82
SPM < 75 μm	91	88	72	78
SPM 75–150 μm	90	86	72	75
SPM 150–250 μm	90	89	65	80

Control: filtered seawater with no microalgae or suspended particulate matter (SPM) added.

## References

[1] Marr J.C., Jackson A.E., McLachlan J.L. (1992). Occurrence of *Prorocentrum lima*, a DSP toxin-producing species from the Atlantic coast of Canada. J. Appl. Phycol..

[2] Dickey R.W., Bobzin S.C., Faulkner D.J., Bencsath Z.F.A., Andrzejewski D. (1990). Identification of okadaic acid from a Caribbean Dinoflagellate, *Prorocentrum concayum*. Toxicon.

[3] Morton S.L., Bomber J.W., Tindall P.M. (1994). Environmental effects on the production of okadaic acid from *Prorocentrum hoffmannianum* Faust I. temperature, light, and salinity. J. Exp. Mar. Biol. Ecol..

[4] An T., Winshell J., Scorzetti G., Fell J.W., Rein K.S. (2010). Identification of okadaic acid production in the marine dinoflagellate *Prorocentrum rhathymum* from Florida Bay. Toxicon.

[5] Kameneva P.A., Efimova K.V., Rybin V.G., Orlova T.Y. (2015). Detection of dinophysistoxin-1 in clonal culture of marine dinoflagellate *Prorocentrum foraminosum* (Faust M.A., 1993) from the Sea of Japan. Toxins.

[6] Reguera B., Velo-Suárez L., Raine R., Park M.G. (2012). Harmful *Dinophysis* species: A review. Harmful Algae.

[7] James K.J., Carey B., O’Halloran J., Van Pelt F.N.A.M., Škrabáková Z. (2010). Shellfish toxicity: Human health implications of marine algal toxins. Epidemiol. Infect..

[8] Valdiglesias V., Laffon B., Pásaro E., Méndez J. (2011). Okadaic acid induces morphological changes, apoptosis and cell cycle alterations in different human cell types. J. Environ. Monit..

[9] Terao K., Ito E., Yanagi T., Yasumoto T. (1986). Histopathological studies on experimental marine toxin poisoning. I. Ultrastructural changes in the small intestine and liver of suckling mice induced by dinophysistoxin-1 and pectenotoxin-1. Toxicon.

[10] Yasumoto T., Oshima Y., Yamaguchi M. (1978). Occurrence of a new type of toxic shellfish poisoning in the Tohoku district. Bull. Jpn. Soc. Sci. Fish.

[11] Li A., Ma J., Cao J., McCarron P. (2012). Toxins in mussels (*Mytilus galloprovincialis*) associated with diarrhetic shellfish poisoning episodes in China. Toxicon.

[12] Trainer V.L., Moore L., Bill B.D., Adams N.G., Harrington N., Borchert J., da Silva D.A.M., Eberhart B.T.L. (2013). Diarrhetic shellfish toxins and other polyether toxins of human health concern in Washington State. Mar. Drugs.

[13] Taylor M., McIntyre L., Ritson M., Stone J., Bronson R., Bitzikos O., Rourke W., Galanis E. (2013). Outbreak Investigation Team. Outbreak of diarrhetic shellfish poisoning associated with mussels, British Columbia, Canada. Mar. Drugs.

[14] MacKenzie L., Holland P., McNabb P., Beuzenberg V., Selwood A., Suzuki T. (2002). Complex toxin profiles in phytoplankton and Greenshell mussels (*Perna canaliculus*), revealed by LC-MS/MS analysis. Toxicon.

[15] Hinder S.L., Hays G.C., Brooks C.J., Davies A.P., Edwards M., Walne A.W., Gravenor M.B. (2011). Toxic marine microalgae and shellfish poisoning in the British Isles: History, review of epidemiology, and future implications. Environ. Health.

[16] Grattan L.M., Holobaugh S., Morris J.G. (2016). Harmful algal blooms and public health. Harmful Algae.

[17] Torgersen T., Wilkins A.L., Rundberget T., Miles C.O. (2008). Characterization of fatty acid esters of okadaic acid and related toxins in blue mussels (*Mytilus edulis*) from Norway. Rapid Commun. Mass Spectrom..

[18] Turner A.D., Goya A.B. (2015). Occurrence and profiles of lipophilic toxins in shellfish harvested from Argentina. Toxicon.

[19] Matsushima R., Uchida H., Nagai S., Watanabe R., Kamio M., Nagai H., Kaneniwa M., Suzuki T. (2015). Assimilation, accumulation, and metabolism of dinophysistoxins (DTXs) and pectenotoxins (PTXs) in the several tissues of Japanese scallop *Patinopecten yessoensis*. Toxins.

[20] Vale P., Sampayo M.A.M. (2002). First conformation of human diarrhoeic poisonings by okadaic acid esters ingestion of razor clams (*Solen marginatus*) and green crabs (*Carcinus maenas*) in Aveiro lagoon, Portugal and detection of okadaic acid esters in phytoplankton. Toxicon.

[21] Jørgensen K., Scanlon S., Jensen L.B. (2005). Diarrhetic shellfish poisoning toxin esters in Danish blue mussels and surf clams. Food Addit. Contam..

[22] García C., Truan D., Lagos M., Santelices J.P., Diaz J.C., Lagos N. (2005). Metabolic transformation of dinophysistoxin-3 into dinophysistoxin-1 causes human intoxication by consumption of *O*-acyl-derivates dinophysistoxins contaminated shellfish. J. Toxicol. Sci..

[23] Doucet E., Ross N.N., Quilliam M.A. (2007). Enzymatic hydrolysis of esterified diarrhetic shellfish poisoning toxins and pectenotoxins. Anal. Bioanal. Chem..

[24] Braga A.C., Alves R.N., Maulvault A.L., Barbosa V., Marques A., Costa P.R. (2016). In vitro bioaccessibility of the marine biotoxin okadaic acid in shellfish. Food Chem. Toxicol..

[25] Torgersen T., Miles C.O., Rundberget T., Wilkins A.L. (2008). New esters of okadaic acid in seawater and blue mussels (*Mytilus edulis*). J. Agric. Food Chem..

[26] European Food Safety Authority (EFSA) (2009). Scientific Opinion of the Panel on Contaminants in the Food Chain on a request from the European Commission on Marine Biotoxins in Shellfish—Summary on regulated marine biotoxins. EFSA J..

[27] MacKenzie L., Beuzenberg V., Holland P., McNabb P., Selwood A. (2004). Solid phase adsorption toxin tracking (SPATT): A new monitoring tool that simulates the biotoxin contamination of filter feeding bivalves. Toxicon.

[28] Rundberget T., Gustad E., Samdal I.A., Sandvik M., Miles C.O. (2009). A convenient and cost-effective method for monitoring marine algal toxins with passive samplers. Toxicon.

[29] Fux E., Bire R., Hess P. (2009). Comparative accumulation and composition of lipophilic marine biotoxins in passive samplers and in mussels (*M. edulis*) on the West Coast of Ireland. Harmful Algae.

[30] Li Z., Guo M., Yang S., Wang Q., Tan Z. (2010). Investigation of pectenotoxin profiles in the Yellow Sea (China) using passive sampling technique. Mar. Drugs.

[31] MacKenzie L. (2010). In situ passive solid-phase adsorption of micro-algal biotoxins as a monitoring tool. Curr. Opin. Biotechnol..

[32] Pizarro G., Moroño Á., Paz B., Franco J.M., Pazos Y., Reguera B. (2013). Evaluation of passive samplers as a monitoring tool for early warning of *Dinophysis* toxins in shellfish. Mar. Drugs.

[33] Li M., Sun G., Qiu J., Li A. (2017). Occurrence and variation of lipophilic shellfish toxins in phytoplankton, shellfish and seawater samples from the aquaculture zone in the Yellow Sea, China. Toxicon.

[34] Li X., Li Z., Chen J., Shi Q., Zhang R., Wang S., Wang X. (2014). Detection, occurrence and monthly variations of typical lipophilic marine toxins associated with diarrhetic shellfish poisoning in the coastal seawater of Qingdao City, China. Chemosphere.

[35] Chen J., Li X., Wang S., Chen F., Cao W., Sun C., Zheng L., Wang X. (2017). Screening of lipophilic marine toxins in marine aquaculture environment using liquid chromatography-mass spectrometry. Chemosphere.

[36] Jauffrais T., Kilcoyne J., Herrenknecht C., Truquet P., Séchet V., Miles C.O., Hess P. (2013). Dissolved azaspiracids are absorbed and metabolized by blue mussels (*Mytilus edulis*). Toxicon.

[37] Song Q., Fang J.G., Liu H., Zhang J.H., Wang L.L., Wang W. (2006). Studies on the effects of suspended sediment on the feeding physiology of three suspension-feeding bivalves. Mar. Fish. Res..

[38] Nielsen L.T., Hansen P.J., Krock B., Vismann B. (2016). Accumulation, transformation and breakdown of DSP toxins from the toxic dinoflagellate *Dinophysis acuta* in blue mussels, *Mytilus edulis*. Toxicon.

[39] Jauffrais T., Contreras A., Herrenknecht C., Truquet P., Séchet V., Tillmann U., Hess P. (2012). Effect of *Azadinium spinosum* on the feeding behaviour and azaspiracid accumulation of *Mytilus edulis*. Aquat. Toxicol..

[40] Kameneva P.A., Imbs A.B., Orlova T.Y. (2015). Distribution of DTX-3 in edible and non-edible parts of *Crenomytilus grayanus* from the Sea of Japan. Toxicon.

[41] Nagai S., Suzuki T., Nishikawa T., Kamiyama T. (2011). Differences in the production and excretion kinetics of okadaic acid, dinophysistoxin-1, and pectenotoxin-2 between cultures of *Dinophysis acuminata* and *Dinophysis fortii* isolated from western Japan. J. Phycol..

[42] Smith J.L., Tong M., Fux E., Anderson D.M. (2012). Toxin production, retention, and extracellular release by *Dinophysis acuminata* during extended stationary phase and culture decline. Harmful Algae.

[43] Alves-de-Souza C., Varela D., Contreras C., LaIglesia P., Fernández P., Hipp B., Hernández C., Riobó P., Reguera B., Franco J.M. (2014). Seasonal variability of *Dinophysis* spp. and *Protoceratium reticulatum* associated to lipophilic shellfish toxins in a strongly stratified Chilean fjord. Deep Sea Res. Part II.

[44] Guillard R.R.L., Hargraves P.E. (1993). *Stichochrysis immobilis* is a diatom, not a chrysophyte. Phycologia.

[45] Li A., Ma F., Song X., Yu R. (2011). Dynamic adsorption of diarrhetic shellfish poisoning (DSP) toxins in passive sampling relates to pore size distribution of aromatic adsorbent. J. Chromatogr. A.

[46] Fan L., Sun G., Qiu J., Ma Q., Hess P., Li A. (2014). Effect of seawater salinity on pore-size distribution on a poly(styrene)-based HP20 resin and its adsorption of diarrhetic shellfish toxins. J. Chromatogr. A.

[47] Li A., Chen H., Qiu J., Lin H., Gu H. (2016). Determination of multiple toxins in whelk and clam samples collected from the Chukchi and Bering seas. Toxicon.

[48] Suzukia T., Otab H., Yamasakia M. (1999). Direct evidence of transformation of dinophysistoxin-1 to 7-*O*-acyl-dinophysistoxin-1 (dinophysistoxin-3) in the scallop *Patinopecten yessoensis*. Toxicon.

[49] Gerssen A., Mulder P.P.J., McElhinney M.A., Boer J. (2009). Liquid chromatography-tandem mass spectrometry method for the detection of marine lipophilic toxins under alkaline conditions. J. Chromatogr. A.

